# Setting research priorities for palliative and end-of-life care: a James Lind Alliance Priority Setting Partnership Refresh

**DOI:** 10.1136/bmjopen-2025-108910

**Published:** 2026-02-12

**Authors:** Briony F Hudson, Phillippa Ashcroft, Jennifer Bedford, Jessica Bush, Ben Bowers, Annabel Dawson, Jamilla Hussain, Sarah Holmes, Rashmi Kumar, Ollie Minton, Angela McCullagh, Louisa Nicoll, Alison Penny, Mary Rabbitte, Alexandra Reece, Diana Robinson, Charlotte Simpson-Greene, Melanie Taylor, Sabine L Best

**Affiliations:** 1 Marie Curie, London, UK; 2 Marie Curie Palliative Care Research Department, University College London Faculty of Brain Sciences, London, UK; 3 Motor Neurone Disease Association, Northampton, UK; 4 Department of Public Health and Primary Care, University of Cambridge, Cambridge, UK; 5 Queen’s Nursing Institute, London, UK; 6 Lived Experience Group, London, UK; 7 Bradford Teaching Hospitals NHS Foundation Trust, Bradford, UK; 8 Wolfson Palliative Care Research Centre, University of Hull, Hull, UK; 9 Marie Curie Research Voices Group, London, UK; 10 National Children’s Bureau, Childhood Bereavement Network, London, UK; 11 All Ireland Institute of Hospice and Palliative Care, Dublin, Ireland; 12 Hospice UK, London, UK

**Keywords:** PALLIATIVE CARE, Research Design, Patient Participation

## Abstract

**Abstract:**

**Background:**

Palliative care supports the physical, emotional, social and spiritual needs of people with serious life-limiting illness. Future research must align with the priorities of people approaching the end of their lives, and those close to them.

**Aims:**

To undertake a refresh of the James Lind Alliance Palliative and End of Life Care Priority Setting Partnership, to identify and prioritise areas for future research.

**Design:**

The James Lind Alliance process was applied, between May 2023 and February 2025. An initial online survey collected areas for future research from participants. These were synthesised into a long list of questions and shortlisted through a second online survey. Final ranking of priorities was achieved using an adapted Nominal Group Technique within a prioritisation workshop.

**Participants:**

People living with serious life-limiting illnesses, carers, friends and family members supporting them, bereaved people, health and social care professionals, volunteers working in palliative and end-of-life care and members of the public.

**Results:**

1032 and 626 responses were received to survey 1 and 2, respectively. 20 people with lived and professional experience attended the prioritisation workshop. An updated list of 24 priorities for palliative and end-of-life care research was produced.

**Conclusion:**

The priorities reflect the range of issues shaping end-of-life experiences and serve as a call to action for researchers and funders.

**STRENGTHS AND LIMITATIONS OF THIS STUDY**

- This study was completed in line with the James Lind Alliance Priority Setting Partnership Guidebook and was overseen by James Lind Alliance Advisors.
- A Steering Group consisting of representatives from a range of health, social care, academic and Lived Experience backgrounds was convened which supported the project for its duration.
- In addition, a separate Lived Experience group, including 20 people with lived experience of either living with or supporting someone with a serious life-limiting condition, also supported the project.
- People with certain characteristics, including men, people from minoritised ethnic backgrounds, people from gender and sexual minority groups and people whose first language was not English were under-represented.

## Introduction

 Palliative care aims to improve the quality of life and death for individuals living with serious life-limiting illnesses by supporting their physical, emotional, social and spiritual needs.^1^ The demand for palliative care is increasing rapidly both across the UK and worldwide. In the UK Nations, around 90% of people nearing the end of life are expected to require some form of palliative support.^2^

International evidence highlights the increasing need for palliative care, particularly in the face of ageing populations,^3^ rising chronic disease burdens^4^ and persistent global inequities.^5^ However, internationally, research in this field receives very little funding compared with other fields of health science research.^6 7^ In 2022, palliative and end-of-life care received only 0.23% of all UK health-related research funding in 2022, just £6.47 million out of a total £2.79 billion.^7^

Given the growing need, and limited research funding in this area, it is essential that future research reflects what is most important to people living with serious life-limiting illness and those supporting them. The James Lind Alliance (JLA) provides an established process for determining research priorities informed by people directly affected by issues. JLA Priority Setting Partnerships (PSPs) bring together patients, families and health and social care professionals to identify and prioritise the ‘top 10’ evidence uncertainties for research to address on set topics.^8^ In 2015, a partnership on palliative and end-of-life care was completed in the UK.^9^ Since the identification of these priorities, differing amounts of research funding have been directed to each of the identified top 10 priorities,^10^ with variation in the progress made in addressing each.

This paper presents the methods and findings from a refresh of the 2015 Palliative and End-of-Life Care Priority Setting Partnership conducted in the UK. The project explored what people with lived and professional experience of palliative and end-of-life care believe should be the key research priorities for the future. These insights could help guide researchers and research funders in shaping the future of palliative and end-of-life care research and improving experiences towards the end of life.

## Methods

**Figure 1:**
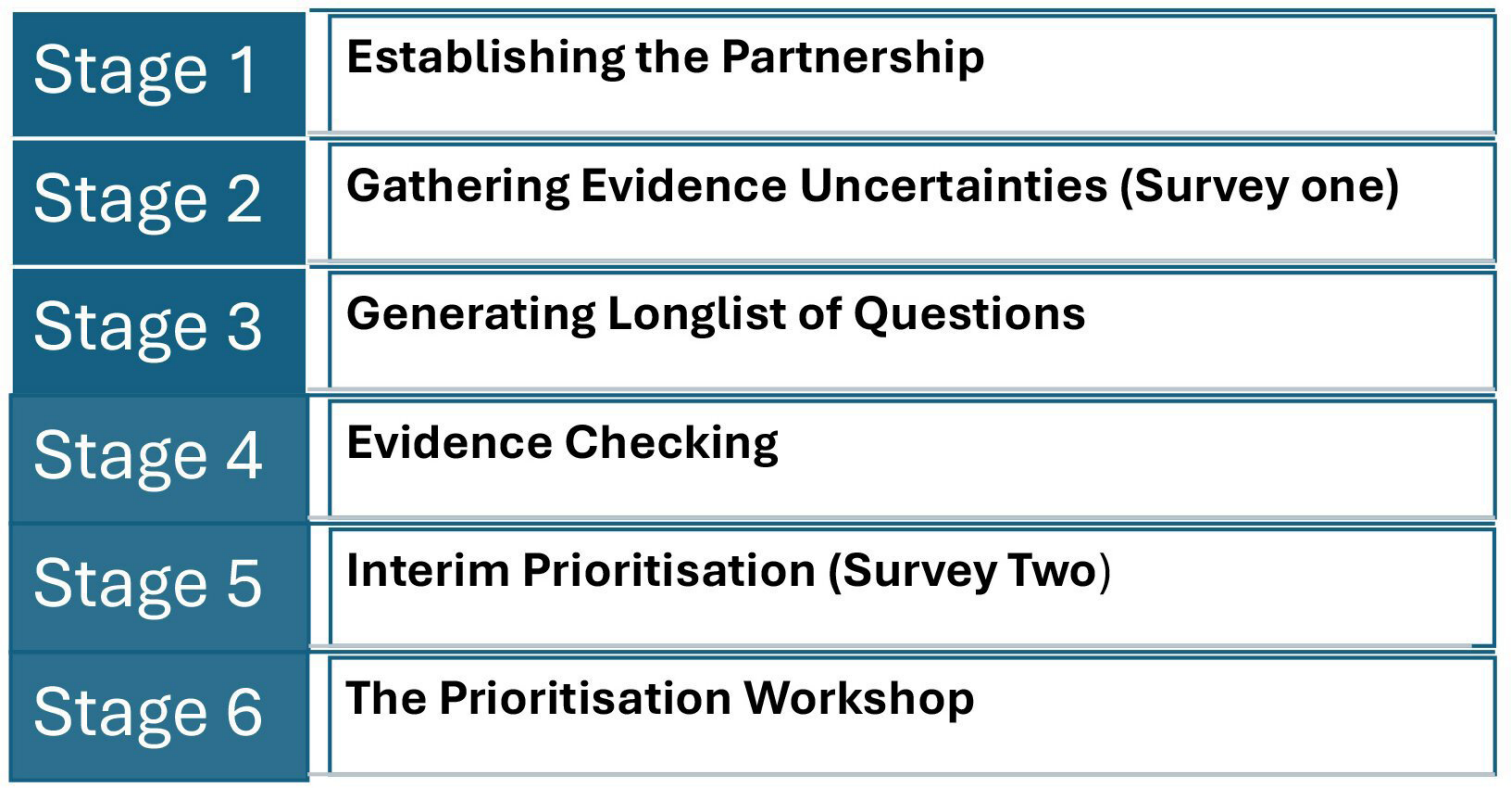
Methodology for the refresh of the James Lind Alliance Palliative and End of Life Care Priority Setting Partnership.

The JLA approach^8^ was created in recognition of the fact that health research agendas have historically been shaped primarily by researchers and pharmaceutical companies. In contrast, the JLA process aims to ensure that people who are typically excluded from research prioritisation, such as those with lived experience, can actively contribute. The process involves two open surveys followed by a prioritisation workshop (figure 1). Typically, a JLA PSP produces a ‘top 10’ list of priorities that highlight important areas for future research.

The project was conducted in line with the JLA guidebook and results are reported in line with the REporting guideline for PRIority SEtting of health research (REPRISE).^11^

### Stage 1: establishing the scope and partnership

The project was overseen by a Steering Group, chaired by senior JLA advisors. Steering Group members included patient and carer representatives (n=6), clinical representatives (n=9, from diverse health and social care backgrounds) and observers from UK health and social care research funders (n=6). A data subgroup, formed of members of the Steering Group, the Lived Experience Group and Marie Curie staff, supported the grouping of data identified in survey 1.

### Step 2: gathering evidence uncertainties (survey 1)

Survey 1 was informed by the initial survey used in the 2015 JLA Palliative and End-of-Life Care Priority Setting Partnership.^12^ This was adapted based on feedback from the Steering Group and Lived Experience Group. The survey included the following open-text question and also gathered demographic data (table 1).

Based on your experiences, what issues should future research address to improve the experience of people with a serious, life-limiting illness and/or those close to them?

**Table 1:** Characteristics of survey respondents and workshop attendees

	Survey 1 n=1032	Survey 2 n=626	Workshop n=20
Gender identity			
Woman	825 (80%)	495 (79%)	16 (80%)
Man	160 (16%)	97 (15%)	4 (20%)
Non-binary	7 (1%)	9 (1%)	0
Prefer not to say	40 (4%)	25 (4%)	0
Sexual orientation			
Bisexual	28 (3%)	14 (2%)	Not collected
Lesbian	15 (1%)	15 (2%)	Not collected
Gay	18 (2%)	7 (1%)	Not collected
Heterosexual	871 (84%)	511 (82%)	Not collected
Other	3 (<1%)	8 (1%)	Not collected
Prefer not to say	97 (9%)	71 (11%)	Not collected
Age	n=1013	n=594	
Range	20–100	22–90	39–75
Mean	53	54	50.5
Ethnicity			
Asian	32 (3%)	14 (2%)	2 (10%)
Black	18 (2%)	7 (1%)	1 (5%)
Mixed	14 (1%)	9 (1%)	0
White	917 (89%)	556 (89%)	17 (85%)
Other	10 (1%)	8 (1%)	0
Prefer not to say	41 (4%)	32 (5%)	0
Religion			
Agnosticism	67 (6%)	50 (8%)	1 (5%)
Buddhism	12 (1%)	17 (3%)	0
Christianity	429 (42%)	246 (39%)	8 (40%)
Hinduism	9 (1%)	6 (1%)	0
Islam	7 (1%)	6 (1%)	1 (5%)
Judaism	8 (1%)	5 (1%)	0
Sikhism	3 (<1%)	0	0
No religion	244 (24%)	143 (23%)	1 (5%)
Other religious or philosophical beliefs*	174 (17%)	92 (15%)	4 (20%)
Prefer not to say	79 (8%)	61 (10%)	5 (25%)
Where do you live			
England	685 (66%)	322 (51%)	17 (85%)
Northern Ireland	76 (7%)	34 (5%)	2 (10%)
Ireland	60 (6%)	3 (<1%)	0
Scotland	96 (9%)	77 (12%)	0
Wales	74 (7%)	62 (10%)	1 (5%)
Living overseas	27 (3%)	23 (4%)	0
Prefer not to say	14 (1%)	105 (17%)	0
Type of lived experience†			
Living with a serious life-limiting illness	92	61	0
Caring for someone with a serious life-limiting illness	192	105	5 (25%)
A bereaved friend or family member	383	213	4 (20%)
Volunteer	62	51	0
Member of the public	156	121	0
Other	42	0	0
Health and social care professional	527	339	11 (55%)
Allied health professional‡	57 (11%)	55 (16%)	1 (9%)
Bereavement specialist	2 (<1%)	4 (1%)	*0*
Care home staff	19 (4%)	4 (1%)	0
Chaplain	10 (2%)	2 (1%)	0
Counsellor/therapist	7 (1%)	2 (1%)	0
Doctor	79 (15%)	48 (14%)	*0*
Frailty practitioner	4 (1%)	0	1 (9%)
Healthcare assistant	27 (5%)	34 (10%)	*0*
Manager of care organisation	66 (13%)	31 (9%)	0
Medical director of care organisation	0	0	1 (9%)
Nurse	168 (32%)	110 (32%)	5 (46%)
Pharmacist	5 (1%)	5 (1%)	1 (9%)
Policymaker	65 (1%)	7 (2%)	0
Psychologist	11 (2%)	1 (<1%)	0
Social worker	21 (4%)	13 (4%)	2 (18%)
Other§/prefer not to say	46 (9%)	23 (7%)	0

* Other religious and philosophical beliefs include spiritual but not religious, humanism, Paganism and Quakerism.

† Includes double counting as respondents could select more than one type of lived experience.

‡ Includes occupational therapists, paramedics, physiotherapists and speech and language therapists.

§ Other professions include doulas, soul midwives, dementia advisors, support workers, social prescribers.

The survey, created in Microsoft Forms, was piloted with several groups including the Steering Group and Lived Experience Group and the Marie Curie Research Voices Group. It was promoted online through partner organisations’ networks, social media, blogs, posters at hospices and an online launch event. The project team also liaised with community and professional groups to support recruitment to the survey. Survey 1 was open from 16 September 2023 to 1 January 2024. People with lived or professional experience were eligible to respond to the survey. Responses from people that identified solely as a researcher were not eligible, in line with the approach outlined in the JLA guidebook.^8^

### Stage 3: generating a longlist of questions

Responses to survey 1 (evidence uncertainties) that were deemed to fall within the scope of this PSP were grouped into categories. The evidence uncertainties in each group were then converted into summary questions by the information specialist. The Data Subgroup, which contained people with lived and professional experience, met online for a total of 12 hours to review and refine these groupings and questions, by checking for similarity between questions, regrouping, rephrasing or excluding questions where necessary.

The longlist of questions was subsequently reviewed and approved by the Steering Group and Lived Experience Group.

### Stage 4: evidence checking

A review of published literature was undertaken to ensure that none of the 79 questions generated in step 3 had been addressed in existing research. In May 2024, PubMed was searched using the terms ‘palliative care’ or ‘bereavement’ and ‘systematic review’ to identify systematic reviews published in English between 2015 and 2024 that related to longlisted questions.^13^ Each question was categorised as being unanswered (if no recent systematic reviews answered the whole question), partially answered (if an existing review addressed a single aspect) or answered (if an existing review addressed all aspects of a question).

If a question was found to have been answered by an existing systematic review, it would not be considered for inclusion in the prioritisation survey.

In August 2023, the information specialist followed the same procedure to identify systematic reviews relating to all 83 questions from the 2015 PSP, to determine whether the priorities identified had since been addressed by research.

### Stage 5: interim prioritisation (survey 2)

Two versions of the interim prioritisation survey were piloted by project team members using Qualtrics. One version asked respondents to rate how much of a priority each longlisted question was to them, on a 4-point Likert scale from ‘very low priority’ to ‘very high priority’, with an alternative ‘don’t know’ option. Longlisted questions were presented in thematic blocks. The order of the blocks and questions was randomised.

In the second version, the full list of 79 priorities was presented and respondents were asked to select a top 10. Feedback from the piloting favoured the first version, which was therefore used to collect responses.

The online interim prioritisation survey was available in English and Welsh and was open between 18 June and 31 August 2024. The survey was distributed in the same way as survey 1 and was open to previous and new participants. It collected the same demographic variables as survey 1. Mean scores for each priority were calculated and ranked. The overall rankings and those based on responses from people with lived experience and people with professional experience were calculated (online supplemental table 2).

### Stage 6: the prioritisation workshop

A prioritisation workshop was held on 5 November 2024 in central London. A sampling framework, which considered age, gender, type of lived experience and location was developed to select and invite participants from previous stages of the project who had expressed an interest in attending the workshop. Attendees were sent an information pack in advance which included the shortlisted 24 questions (those which received the highest rankings in the analysis of survey 2 data), in a random order, and were asked to either rank all the questions, or identify their top and bottom three. Three independent JLA facilitators used an adapted Nominal Group Technique to generate discussion, ranking, consensus and agreement between attendees^8^ and produce the final ranking of priorities (table 2).

**Table 2:** Identified priorities for palliative and end-of-life care research

Priority number	Question
1	How do *people with dementia* experience end of life? How can palliative and end-of-life care better meet their needs and those of their carers, friends and families?
2	How can NHS, social services and charities work more *collaboratively to provide joined-up care* that better meets the needs of people with a serious life-limiting illness and their carers, friends and families?
3	What kinds of palliative and end-of-life care and support need to be in place *to enable people to die well at home*? What skills do staff need? What helps or hinders the delivery of care at home?
4	What are the best ways to provide *personalised palliative and end-of-life care* that meets all the physical, mental, practical, social and spiritual needs of a person with a serious life-limiting illness?
5	How can palliative and end-of-life care better meet the complex needs of people with *multiple health conditions*?
6	How can *communication and care co-ordination be improved* across the teams of health and social care professionals caring for people with any serious life-limiting illness?
7	What *skills, training and information do carers, friends and family members* need to be able to care for someone who is dying at home (eg, giving medicines safely by injection, managing incontinence, moving people)? What is the impact (pros and cons) of upskilling the people giving care?
8	How can the *quality of palliative and end-of-life care in hospital* be improved? What helps or hinders improvement?
9	What are the best ways to provide palliative and end-of-life care, *support and advice at all hours* (24/7 or out of hours)?
10	How can palliative and end-of-life care better meet the needs of people *who live alone, or are socially isolated*?
11	What are the best ways to *train and support staff* who provide care at the end of life—either at home, in care homes or nursing homes?
12	How can it be ensured that *everyone has access to the palliative* *and end-of-life care they want or need*?
13	How can palliative and end-of-life care better meet the *needs of people with illnesses other than cancer*, eg, chronic obstructive pulmonary disease, pulmonary fibrosis, organ failure, motor neuron disease or chronic fatigue syndrome?
14	What are the best ways to *identify that a person is dying or is near to death* (in their last year, months, weeks, days of life)? How can health professionals better recognise these stages in people with any serious life-limiting illness?
15	What are the best ways to provide *emotional and psychological support to people with a serious life-limiting illness*, from diagnosis through to end of life?
16	What are the best ways to *prepare carers, friends and families for what will happen* while the person they care for is dying? What information about symptoms during the last few days and hours is helpful to know?
17	How can *symptoms* at the end of life be *managed in a timely way*, including by *rapid access to medicines*?
18	What are the best ways to manage people who are *restless, agitated or experience sudden confusion* (delirium) at the end of life?
19	What *stops people’s wishes from being acted on* (eg, with future or advance care plans)? How can any barriers be overcome?
20	How can *discharge from hospital* be improved for people with any serious life-limiting illness?
21	What are the best ways to provide *palliative and end-of-life care in the community*, for example, what are the roles of different services and professions?
22	What are the best ways to ensure *adequate pain relief* for people with a serious life-limiting illness?
23	What are the *best ways to provide support to children* when someone important to them is dying, or has died (eg, psychological and social support)?
24	Can *medicines* be changed so that they can be *more easily administered at home* and in the *community* at end of life, eg, through injection under the skin, or under the tongue?

NHS, National Health Service.

### Patient and public involvement

A Lived Experience Group including people with life-limiting conditions (n=1), bereaved family members (n=17) and informal carers (n=2) across the four nations of the UK was established to advise and support the PSP. The group met online nine times between May 2023 and November 2024. The Public Involvement in Research Impact Tool^12^ was used to capture their impact alongside semi-structured interviews and a feedback survey. A paper outlining the impact of the group is published separately.^14^

## Results

### Stage 1: establishing the scope and partnership

The partnership agreed that the PSP’s scope should focus on experiences of adults living with a serious, life-limiting illness and of dying, death and bereavement, including palliative and end-of-life care. Issues related to people younger than 18, curative or prevention treatment were outside of the scope of this PSP.

### Stage 2: gathering evidence uncertainties (survey 1)

There were 1046 responses to survey 1. No requests to complete the survey in a different language or over the phone were received. Seven responses were excluded as respondents identified as researchers only and a further seven were excluded as the open text response box was left blank.

1032 responses were included in the analysis. Respondents included people living with a serious life-limiting illness (n=92), people currently caring for someone with a serious life-limiting illness (n=193), bereaved friends or family members (n=386), health and social care professionals (527) (table 1).

The mean age of the sample was 53 years (ranging from 20 to 100). Most respondents were women (80%), identified as heterosexual (84%) and white (89%).

### Stage 3: summarising results gathered

From the 1032 included responses, 2052 evidence uncertainties were identified. These were reviewed by the information scientist and the data subgroup. After discussion with the data subgroup, 418 evidence uncertainties were removed, leaving 1634 to be reviewed. The in-scope responses were then grouped into nine different areas: bereavement, informal carers, prognosis and communication, health services, tailored care, support, treatment, psychological and social support, advance care plans.

This process resulted in a list of 79 questions to be carried forward to the next stage of the project.

### Stage 4: evidence checking

After reviewing the literature, 292 reviews relating to the 79 longlisted questions generated through survey 1 were identified. None had been fully addressed by research, while one had been partially addressed (relating to communicating uncertainty^13^).

464 reviews relating to the priorities identified in the 2015 PSP were identified. None had been fully answered by reviews, while 20 questions had a partial answer.^13^

### Stage 5: interim prioritisation (survey 2)

In total, 626 responses were included in the analysis of survey 2 (table 1), including people living with a serious life-limiting illness (n=61), informal carers (n=105), bereaved friends or family members (213) and health and social care professionals (329). Four respondents completed the survey in Welsh, and two paper copies were returned. The average age of respondents was 54 (range 22–90). The demographic profile of respondents was broadly similar to that of survey 1.

There was a high level of agreement between people with lived experience and those with professional experience with regards to the prioritisation of the issues. Issues with less agreement included questions relating to barriers to advance care plans being acted on (ranked 6th by professionals and 30th by people with lived experience), meeting the needs of people with multiple health conditions (ranked 20th by professionals and 33rd by people with lived experience) and managing restlessness, agitation or delirium (ranked 8th by people with lived experience and 24th by professionals).

The full rankings of each longlisted questions can be found in online supplemental table 2. The 20 questions with the highest rankings from people with lived experience and those with professional experience resulted in a list of 24 questions, which were discussed at the final prioritisation workshop.

### Stage 6: prioritisation workshop

20 people with a range of experiences relating to palliative care attended the workshop, including people currently caring for someone with a serious life-limiting illness (n=5), bereaved friends or family members (n=4) and health and social care professionals (n=11) (table 1). The mean age of attendees was 50.4 years (range 39–75).

Most workshop participants were women (n=16, 80%), white ethnicity (n=17, 85%) and from England (n=17, 85%), although Wales (n=1, 5%) and Northern Ireland (n=2, 10%) were represented (table 1). A specialist counsellor was available to provide support on the day.

Table 2 outlines the top 24 priorities for palliative and end-of-life care research as ranked by workshop participants.

### Sharing the priorities

The identified priorities were shared at a Research Summit in London in February 2025, attended by researchers, research funders, policymakers and people with lived experience from across the UK. The project was also presented at the Marie Curie Research Conference,^15^ the Hospice UK conference^16^ and the European Association of Palliative Care Conference.^17^ A full and summary report^18^ was designed by Marie Curie and included in newsletters of partner organisations.

## Discussion

The priorities for future palliative and end-of-life care identified through this refresh of a JLA PSP include long-standing systemic issues such as access to support outside of working hours,^19 20^ poor communication^21 22^ and limited continuity of care.^23^ These challenges are well known yet remain inadequately addressed through research and practice. The inclusion of these recognised issues serves as a reminder of what is important to people with lived experience and reinforces the need for further research funding for solution-focused research, evaluation and translation work in these areas.

An evidence check of the 2015 Palliative and End of Life Care PSP completed as part of this project found that none of the original priorities had been fully addressed in existing systematic reviews. However, a grant mapping analysis indicated that each priority had received at least some UK research funding since 2015.^10^ The top 2015 priority, improving care outside of working hours received £1.6 million across 11 grants, yet remains at priority number nine in 2025, highlighting the need for further research, investment and stronger 24/7 care models.

Caring for people with dementia, the top priority in the 2025 PSP, was also identified in 2015, where it ranked 14th. New 2025 priorities not present in the 2015 list include developing effective personalised palliative care (priority 4) and improving care for people with multiple health conditions (priority 5).

The identified priorities align with other international prioritisations for palliative care research. A systematic review of international literature relating to research priorities for palliative and end-of-life care^24^ identified seven priority areas: service models, continuity of care; training and education; equality of access; communication; patient preference and experience; and family carers. The 24 priorities identified in the current PSP align well with these priority areas. However, additional issues were present in this PSP that were not highlighted in the international review, including support for children when someone close to them is dying, care in and discharge from hospital, support for people who live alone, barriers to expressed wishes being acted on, emotional and psychological support for patients and managing delirium at the end of life.

The priorities identified in this PSP also echo aspects of palliative care championed in the Ambitions Framework for England.^25^ For example, treating people as individuals (priority 4), fair access to care (priority 12), maximising comfort and well-being (priority 15, 17, 18, 22, 24), coordinating care (priority 2), ensuring all staff (priority 11) and each community is prepared to care (priority 16) and involving, supporting and caring for those important to the dying person (priority 23).

Palliative care promotes physical, mental, spiritual and social aspects of well-being for both people living with serious life-limiting illness and those close to them. The holistic nature of palliative care is one of its strengths but is also what poses a challenge for the identification of neat priorities for future research.

The priorities identified through this project constitute broad thematic areas for investigation rather than discrete research questions. Addressing these priorities effectively necessitates robust interdisciplinary collaboration to support the development of high-quality research capable of enhancing end-of-life care. Each discipline contributes distinct methodological approaches, theoretical frameworks and interpretive lenses, all of which enrich the collective understanding of dying, death and bereavement. Expanding scholarly discourse in these areas is both necessary and strongly encouraged.^26^

### Strengths and limitations

Both surveys collected demographic data from respondents, something which is not consistently done across PSPs. This data highlighted how certain groups, including men, people from minoritised ethnic backgrounds, people from gender and sexual minority groups and people whose first language was not English were under-represented. Other PSPs which collected demographic data seem to have experienced similar challenges in recruiting a diverse sample.^27^ To try and reach as wide a sample as possible we offered the option of receiving paper copies of the surveys, large print versions of the survey, completing them over the phone and signposted to resources to support completion of the survey in multiple languages (via Microsoft Forms). Consideration of which methods might be most suitable for reaching diverse samples should be considered in future PSPs.

While the JLA methodology is a transparent and rigorous process it has some limitations.^28^ In this project, the data subgroup wrestled with the level, or lack of interpretation that could be applied to data collected through the initial survey. Several responses to the initial survey were vague or very brief, for example, ‘symptom management’. In line with the JLA approach, statements such as this were considered too broad, as the issue underlying the response could not be determined. The timely management of symptoms was included in the final list but specific questions about some individual symptoms were not, despite appearing in survey responses.

Another point to note is the change in rankings of priorities between the prioritisation survey (survey 2) and the final set of priorities agreed on at the final workshop. For example, among the 626 responses to the prioritisation survey, exploring how to ensure adequate pain relief for people with a serious life-limiting illness was the issue ranked of highest importance. This issue appears at number 22 within the final list of 24 priorities. Adequate pain management is a key component of dying well,^29^ and being free from pain and other symptoms has been reported as the public’s top wish for the end of their life.^30^

This PSP was guided by a Steering Group made up of patient and carer representatives (n=6), five from the Lived Experience group and one from the Marie Curie Research Voices group, professionals from diverse health and social care backgrounds (n=9) and representatives from health and social care research funders across all UK nations (n=6). The group was chaired by a senior JLA adviser to ensure full adherence to JLA principles. Working in parallel with the Steering Group, the Lived Experience group and the JLA advisory team helped maintain transparency and rigour throughout the project.

### Impact of the Lived Experience Group

A Lived Experience Group was embedded within this project, alongside the Steering Group. Engagement with the Lived Experience Group provided valuable insights that informed and strengthened the project.^14^

Within Lived Experience Group Meetings, there was much discussion around the language used in the project. For example, there was much discussion around the use of the word ‘death’ within survey 1. Some members felt this word could be a deterrent to survey completion. Terms such as ‘death’ and ‘dying’ can be experienced as blunt but are useful for promoting the transparency needed for future planning, especially towards the end of life.^31 32^ Openness and reactions to language around death and dying are influenced by a myriad of factors including proximity to death,^33^ death literacy,^34^ gender,^35^ age^36^ and religiosity.^37^ It was interesting to reflect on this as a group and consider carefully the language used in the project.

Input from the group within the data subgroup challenged us to reconsider the process of deciding which responses to survey 1 could be considered ‘out of scope’. Following input from the group, discussions on this issue were deferred until data collection was complete.

The group also championed promotion of the top 24 priorities, rather than only the top 10, as is usual for priority setting partnerships. Lived Experience Group members wanted to ensure that issues such as pain, equity and timely symptom treatment, which ranked in the top 10 of survey 2 but not in the workshop’s top 10, were also considered in the development and funding of future research.

### Implications for future research

Researchers from a range of disciplines are encouraged to explore and build on this work by applying different disciplinary lenses and forging new collaborations. Promoting the refreshed priorities within and outside of palliative care for both researchers and funders will be important. This has begun within the social sciences and humanities^26^ and mental well-being^38^ research fields and others could consider how their methods and perspectives could respond to identified priorities.

Further research could explore whether these priorities resonate with groups that were under-represented in this study, differ in international contexts, or if different methods of engagement and data collection could impact the diversity of respondents. More targeted research involving broader demographic inclusion, or focusing on the priorities that may be identified by people from groups that are under-represented in the current study would be beneficial and could provide insights to address inequity in palliative care.

## Conclusions

The propensity of palliative care to consider the whole person, their environment, preferences, circumstances and relationships is what makes the discipline unique but challenges the identification of neat research priorities. The issues represented here are broad, reflecting the diverse range of issues that shape end-of-life experiences. Work remains to identify specific research questions within these priorities, and the right approaches to tackling them. Greater investment in palliative care research will be needed to respond to these identified needs and to ensure that the voices of those who are often under-represented in research are heard.

## Supplementary material

10.1136/bmjopen-2025-108910 online supplemental file 1

## Data Availability

Data are available in a public, open access repository.
