# Supplementary material for: Setting research priorities for palliative and end-of-life care: a James Lind Alliance Priority Setting Partnership Refresh
Source: BMJ Open. 2026 Feb 12;16(2):e108910. doi: 10.1136/bmjopen-2025-108910 (PMC12911823; doi:10.1136/bmjopen-2025-108910)
Supplement: online supplemental file 1 [file bmjopen-16-2-s001.docx]

*Table 2. Overall rankings of longlist of priorities for different groups of respondents within the interim survey*

| ***Question*** | ***n*** | ***Overall average*** | ***Overall ranking*** | ***People with lived experience ranking*** | ***Health and social care professionals ranking*** |
| --- | --- | --- | --- | --- | --- |
| What are the best ways to ensure adequate **pain relief** for people with a serious life-limiting illness? | *610* | *3.63* | *1* | *1* | *8* |
| How can it be ensured **that everyone has access** to the palliative and end of life care they want or need? | *619* | *3.61* | *2* | *2* | *2* |
| What kinds of palliative and end of life care and support need to be in place to **enable people to die well at home?** What skills do staff need? What helps or hinders the delivery of care at home? | *616* | *3.58* | *3* | *4* | *1* |
| How can **symptoms at the end of life be managed in a timely way**, including by rapid access to medicines? | *611* | *3.55* | *4* | *3* | *9* |
| How can NHS, social services and charities work more collaboratively to provide **joined-up care** that better meets the needs of people with a serious life-limiting illness and their friends and families? | *611* | *3.55* | *5* | *5* | *4* |
| What are the best ways to provide palliative and end of life care, support and advice at all hours (**24/7 or out of hours**)? | *616* | *3.53* | *6* | *7* | *3* |
| What are the best ways to provide **support (e.g. psychological and social support) to children** when someone important to them is dying, or has died? | *606* | *3.49* | *7* | *9* | *5* |
| How can **discharge from hospital** be improved for people with any serious life-limiting illness? | *605* | *3.49* | *8* | *12* | *7* |
| How can palliative and end of life care better meet the needs of people with **illnesses other than cancer** e.g. chronic obstructive pulmonary disease (COPD), pulmonary fibrosis, organ failure, motor neuron disease (MND) or chronic fatigue syndrome (CFS)? | *595* | *3.48* | *9* | *13* | *11* |
| How can **communication and care co-ordination** be improved across the teams of health and social care professionals caring for people with any serious life-limiting illness? | *614* | *3.47* | *10* | *6* | *13* |
| What **skills, training and information do carers, friends and family members need** to be able to care for someone who is dying at home (e.g. giving medicines safely by injection, managing incontinence, moving people)? What is the impact (pros and cons) of upskilling the people giving care? | *614* | *3.47* | *11* | *10* | *10* |
| How can the quality of **palliative and end of life care in hospital** be improved? What helps or hinders improvement? | *603* | *3.44* | *12* | *15* | *15* |
| What are the best ways to **prepare carers**, friends and families for what will happen while the person they care for is dying? What information about symptoms during the last few days and hours is helpful to know? | *618* | *3.44* | *13* | *11* | *19* |
| How do people with **dementia** experience end of life? How can palliative and end of life care better meet their needs and those of their carers, friends and families? | *605* | *3.43* | *14* | *21* | *12* |
| What are the best ways to **manage people who are restless, agitated** or experience sudden confusion (delirium) at the end of life? | *601* | *3.43* | *15* | *8* | *24* |
| How can palliative and end of life care better meet the needs of **people who live alone, or are socially isolated**? | *617* | *3.42* | *16* | *20* | *14* |
| Can medicines be changed so that they can be more **easily administered at home** and in the community at end of life e.g. through injection under the skin, or under the tongue? | *604* | *3.42* | *17* | *14* | *25* |
| What are the **best ways to train and support staff** who provide care at the end of life - either at home, in care homes or nursing homes? | *612* | *3.42* | *18* | *17* | *18* |
| What are the best ways to **identify that a person is dying or is near to death** (in their last year, months, weeks, days of life)? How can health professionals better recognise these stages in people with any serious life-limiting illness? | *616* | *3.41* | *19* | *19* | *17* |
| What are the best ways to provide **emotional and psychological support** to people with a serious life-limiting illness, from diagnosis through to end of life? | *615* | *3.39* | *20* | *16* | *27* |
| What are the best ways to provide palliative and end of life **care in the community,** for example what are the roles of different services and professions? | *610* | *3.39* | *21* | *31* | *16* |
| What should be included in **initial and ongoing training** to make sure all health professionals across different health care settings can provide palliative and end of life care? | *602* | *3.38* | *22* | *23* | *23* |
| What are the best ways to provide personalised palliative and end of life care that meets all the **physical, mental**, **practical, social and spiritual needs** of a person with a serious life-limiting illness? | *614* | *3.38* | *23* | *18* | *26* |
| How can palliative and end of life care better meet the needs of people who have **communication difficulties** (e.g. people with learning disabilities, or people who have lost their speech or language)? | *606* | *3.37* | *24* | *29* | *21* |
| When and how can people with a serious life-limiting illness best **be supported to make decisions** about their future care, e.g. advance care planning? | *621* | *3.36* | *25* | *24* | *48* |
| What are the best ways to **promote well-being and quality of life** for people at the end of life? | *610* | *3.35* | *26* | *25* | *33* |
| What practical support do carers of people with a serious life-limiting illness need to **maintain their own health and well-being** (e.g. respite care)? | *620* | *3.35* | *27* | *22* | *34* |
| How can palliative and end of life care better meet the complex needs of people with **multiple health conditions**? | *612* | *3.35* | *28* | *33* | *20* |
| When is the **best time to start** delivering palliative and end of life care for any serious life-limiting illness? What is the impact of the timing of referral on the experience of end of life? | *611* | *3.34* | *29* | *37* | *22* |
| How can **communication** between staff delivering care at the end of life and informal carers, friends and families be improved? | *621* | *3.33* | *30* | *27* | *35* |
| How can health professionals, people with a serious life-limiting illness and their carers, friends and families be supported to make decisions about **when to stop clinical tests and treatments**? | *606* | *3.33* | *31* | *26* | *37* |
| What are the best ways to meet the palliative and end of life care needs of **teenagers/ young adults moving into adult services?** | *589* | *3.33* | *32* | *36* | *31* |
| What **stops people’s wishes from being acted upon** (e.g. with future or advance care plans) How can any barriers be overcome? | *615* | *3.32* | *33* | *30* | *6* |
| How can the public’s and health professionals’ **understanding of palliative and end of life care** be improved? | *617* | *3.31* | *34* | *43* | *29* |
| What helps people **come to terms** with the end of their life? | *608* | *3.30* | *35* | *35* | *36* |
| What are the best ways to help carers, friends and **family members to communicate with and support** the person who is dying? | *613* | *3.30* | *36* | *32* | *44* |
| What is the role of **sedation** in palliative and end of life care? What are the best ways to make decisions about this treatment? | *585* | *3.30* | *37* | *28* | *46* |
| What is the role of **specialist palliative care services** and how are they best provided? What are the benefits of specialist palliative care and who benefits the most? | *605* | *3.28* | *38* | *42* | *38* |
| What barriers do health professionals face in having open**, honest and sensitive conversations about death and dying**, including communicating uncertainty? | *614* | *3.27* | *39* | *45* | *32* |
| What are the best ways to encourage and support people in the UK **to talk about death and prepare for death** much earlier? | *617* | *3.26* | *40* | *54* | *28* |
| How can palliative and end of life care better meet the needs of people who are **frail, including people who are very old**? | *609* | *3.25* | *41* | *39* | *43* |
| How can palliative and end of life care better meet the needs of people who have **mental health problems, trauma, addiction, are in prison or experiencing homelessness**? | *586* | *3.25* | *42* | *53* | *30* |
| How can it be determined if carers, friends or family members are **able to take on the caring role** for a person with a serious life-limiting illness, particularly as the person’s symptoms get worse? | *614* | *3.25* | *43* | *34* | *55* |
| When is the **best time to start discussions** about palliative and end of life care with people with any serious life-limiting illness? | *610* | *3.25* | *44* | *48* | *39* |
| What are the best ways to manage **nausea and vomiting** for people with a serious life-limiting illness? | *594* | *3.24* | *45* | *38* | *50* |
| How can **carers, friends and family members be more involved in decisions** about palliative and end of life care for the person they care for? | *620* | *3.22* | *46* | *41* | *54* |
| What are the best ways to provide **emotional and psychological support to carers, friends and families** of someone with a serious life-limiting illness? | *624* | *3.22* | *47* | *46* | *45* |
| What is important and useful to measure (**outcome measures**) when assessing the quality and effectiveness of palliative and end of life care? | *604* | *3.21* | *48* | *58* | *41* |
| What are the best ways to provide **support (e.g. psychological and social support) to adults** when someone important to them is dying, or has died? | *614* | *3.20* | *49* | *47* | *47* |
| How can palliative and end of life care better meet the needs of people with are **neurodiverse** (autistic people or people with attention deficit disorder)? | *582* | *3.20* | *50* | *55* | *42* |
| What do people consider to be **a good death**? What are the best ways to achieve this? | *610* | *3.20* | *51* | *49* | *53* |
| What are the best ways to help people quickly and easily f**ind information about available palliative and end of life care services,** and access those they need? | *616* | *3.19* | *52* | *44* | *58* |
| What are the best ways to help people with a serious life-limiting illness who are having **problems with eating and drinking**? | *601* | *3.19* | *53* | *40* | *62* |
| Which ways of **organising services** have the best outcomes in terms of sustainable improvements in patient experience at the end of life? | *587* | *3.18* | *54* | *50* | *52* |
| How might palliative care services need **to adapt to demographic changes** such as the aging population? | *599* | *3.17* | *55* | *60* | *40* |
| How can patients and people with **lived experience best be included** in palliative and end of life care research? | *605* | *3.16* | *56* | *51* | *59* |
| What are the **emotional impacts on staff** in contact with people receiving palliative and end of life care? What are the best ways to support those staff? | *606* | *3.15* | *57* | *61* | *49* |
| What are the best ways to provide **financial support to people at the end of life** and their carers, friends and families? | *611* | *3.14* | *58* | *52* | *60* |
| What are the best ways to manage **chest secretions** at the end of life? | *557* | *3.12* | *59* | *56* | *61* |
| What are the best approaches to, and benefits of physical support such as physiotherapy and occupational therapy (**palliative rehabilitation**) for people with a serious life-limiting illness? | *596* | *3.11* | *60* | *62* | *57* |
| How can palliative and end of life services best meet the needs of people with a serious life-limiting illness with **different faiths, ethnic backgrounds, languages and cultures**? | *605* | *3.10* | *61* | *67* | *51* |
| What happens to carers, friends and family members when the person they cared for dies, and their caring role ends? What are the **long-term impacts of having cared for someone** with a life-limiting illness? | *613* | *3.08* | *62* | *63* | *63* |
| What are the best ways to support people who experience long-lasting grief (**prolonged grief disorder**)? | *598* | *3.07* | *63* | *65* | *56* |
| How can the severity of a person’s condition be measured in hospice settings **to decide what level of care they need**? | *571* | *3.05* | *64* | *59* | *69* |
| What influences people’s **experiences of pre-bereavement and bereavement** e.g. lack of counselling, financial insecurity, trauma? | *596* | *3.03* | *65* | *68* | *64* |
| Are people with a serious life-limiting illness appropriately treated when they have **other health problems** e.g. bone fractures? | *569* | *3.03* | *66* | *64* | *67* |
| What are the differences in costs and quality of care (outcomes) for people receiving **in-patient hospice care versus people being supported at home**? | *591* | *3.02* | *67* | *66* | *65* |
| What are the best ways to manage **incontinence** at the end of life? | *593* | *3.00* | *68* | *57* | *72* |
| What difference do **palliative care social workers make**? | *555* | *2.90* | *69* | *70* | *70* |
| When should liquid foods and drinks delivered through a tube be given at the end of life (**artificial nutrition and hydration**)? | *555* | *2.90* | *70* | *69* | *74* |
| How can palliative and end of life care better meet the needs of people from **LGBTQ+ communities**? | *574* | *2.89* | *71* | *75* | *66* |
| Do **complementary therapies** (e.g. acupuncture, massage, cannabis oil, creative therapies etc) benefit people with a serious life-limiting illness in terms of well-being, quality of life and/or managing symptoms? | *589* | *2.86* | *72* | *71* | *71* |
| What are the best ways to provide **spiritual support** to people with a serious life-limiting illness and their carers, friends and families? What are the benefits? | *594* | *2.84* | *73* | *74* | *68* |
| What difference do **volunteers** make to the experience of people with serious life-limiting illness and their carers? | *586* | *2.78* | *74* | *73* | *73* |
| Are steroids an effective treatment of **blocked bowels** in people with a serious life-limiting illness? | *443* | *2.76* | *75* | *72* | *75* |
| How useful are **digital interventions** (e.g. apps, remote monitoring at home) in palliative and end of life care? | *570* | *2.66* | *76* | *77* | *76* |
| What are the benefits of **interactions with animals** at the end of life, including contact with pets? | *595* | *2.64* | *77* | *76* | *77* |
| What difference do **doulas** make to the experience of end of life (a non-medically trained person providing a range of support)? How do they work with health professionals? | *508* | *2.50* | *78* | *78* | *79* |
| How can palliative and end of life care be delivered in a more **environmentally sustainable way**? | *593* | *2.46* | *79* | *79* | *78* |

*Includes people living with a serious life-limiting illness, informal carers, people who have been bereaved, volunteers
