# Supplementary material for: Setting research priorities for palliative and end-of-life care: a James Lind Alliance Priority Setting Partnership Refresh
Source: BMJ Open. 2026 Feb 12;16(2):e108910. doi: 10.1136/bmjopen-2025-108910 (PMC12911823; doi:10.1136/bmjopen-2025-108910)
Supplement: Supplementary file 2 [file bmjopen-2025-108910.reviewer_comments.pdf]

PEER REVIEW HISTORY

BMJ Open publishes all reviews undertaken for accepted manuscripts. Reviewers are asked to complete a checklist review form and are provided with free text boxes to elaborate on their assessment. These free text comments are reproduced below.

ARTICLE DETAILS

Title (Provisional)

Setting Research Priorities for Palliative and End of Life Care: A James Lind Alliance Priority Setting Partnership Refresh

Authors

Hudson, Briony F; Ashcroft, Phillippa; Bedford, Jennifer; Bush, Jessica; Bowers, Ben; Dawson, Annabel; Hussain, Jamilla; Holmes , Sarah; Kumar , Rashmi; Minton, Ollie; McCullagh, Angela; Nicoll, Louisa; Penny, Alison; Rabbitte, Mary; Reece, Alexandra; Robinson , Diana; Simpson-Greene, Charlotte; Taylor , Melanie; Best, Sabine L

VERSION 1 - REVIEW

**Reviewer**	**1**
**Name**	**Bayly, Joanne**
**Affiliation**	**King's College London, Cicely Saunders Institute for Palliative Care, Policy and Rehabilitation**
**Date**	**17-Sep-2025**
**COI**	**None**

Thank you for submitting this manuscript for publication which updates research priorities in palliative care and will support development of research proposals and grant applications.

A few very minor points.

In 'What is already known' section, you describe top 11 priorities in 2015 PSP. In the introduction you describe top 10 priorities.

Unsure why responses from researchers were excluded given many working full time in pall care research were once clinicians and have experience. Were researchers only excluded if they had no clinical or professional experience in palliative care? This may need clarifying.

Given researchers employed by funder, could strengths and limitations section describe what means were taken to ensure transparency of or limits on funder influence on the findings.

Grammar and Typos:

Page 6, Lines 14-16. "Responses could contain relate to..." sentence needs reviewing.

Page 10, line 53 review sentence "None been fully addressed by research"

Page 14, line 35-38 review "Several responses to the initial survey were vague or very brief. For example, “symptom management” was detailed." - was symptom management detailed or is this an example of a vague response?

**Reviewer**	**2**
**Name**	**Coyle, Seamus**
**Affiliation**	**University of Liverpool, MCPCIL**
**Date**	**11-Nov-2025**
**COI**	**None**

I have no comments

**Reviewer**	**3**
**Name**	**Chong, P**
**Affiliation**	**HCA Hospice Care, Singapore**
**Date**	**13-Dec-2025**
**COI**	**None**

This is important work. The paper reads well.

I share a few points that might make it even better. For your consideration please.

The section after the abstract appears to fit another journal’s house style. Potentially revise to fit this journal’s requirements. I recall it is about “Strengths and limitations of this study.”

Under the method section, I wonder if something could be said about the James Lind Alliance approach? Figure 1 does not say much honestly. How is this different from other approaches and what are its merits, for example. This helps orient readers (like me) who may be piqued but don’t have time to read the extensive guide cited. After all, this is THE study design adopted here.

Line 40/41 on page 4 is missing a full stop. At first I thought children were in, but they are excluded.

Line 15/16 on page 5. Is a word missing there between “contain” & “relate”? Same for line 39/40 same page: “Two versions …” AND “piloted XXX project”?

I am wondering if Table 2 could be made easier to take in. As is, it looks like a massive spreadsheet that demands careful analysis. Shortly after comes Table 3. In contrast, that seems critical and bold fonts used help to signpost pertinent points quickly.

The discussion section appears somewhat brief. It could be expanded to explore how, where and why priorities here differ from the previous list ten years ago. Given that the procedures involved were carried out in the UK among stakeholders there, does it mean findings apply only to commissioners or clinician-researchers there? The quick mention of how questions align with the international research priorities paper by Hasson et al could be broadened too, with comments on differences, for example, given the unique methodology you adopted here.

I appreciate the opportunity to review this paper. Hope my comments are helpful.

Reviewer 4

Name Winstanley, Emma

Affiliation NHS Blood and Transplant Tissue and Eye Services, UK National Health Service Blood and Transplant Services- Organ and Tissue Donation and Transplantation (NHS BT – OTDT)

Date 23-Dec-2025

COI None

My comments relate to the limitations of the respondent groups being predominantly white women. Although this is acknowledged in the paper, this and the very broad topics, could suggest that rather than a repeat, refresh of the original, more targeted future research involving broader demographic inclusion, harder to reach groups and patients at the end of life could support these initial findings?

VERSION 1 - AUTHOR RESPONSE

Comment	Response
**Reviewer 1 - Dr. Joanne Bayly, King's College London**	
In 'What is already known' section, you describe top 11 priorities in 2015 PSP. In the introduction you describe top 10 priorities.	This section has now been removed.
Unsure why responses from researchers were excluded given many working full time in pall care	Thanks for highlighting this point. In line with the JLA guidebook, researchers were excluded in the prioritisation process. The JLA have taken this position, as historically, the health research agenda

research were once clinicians and have experience.<br><br>Were researchers only excluded if they had no clinical or professional experience in palliative care? This may need clarifying	has been largely determined by the pharmaceutical industry and researchers themselves. The purpose of the JLA process is to enable people with lived experience and health and social care professionals to share their views on what the focus of future research should be.<br><br>The following has been added to the methods section to clarify this:<br><br>“People with lived or professional experience were eligible to respond to the survey. Responses from people that identified solely as a researcher were not eligible, in line with the approach outlined in the JLA guidebook (8).”
Given researchers employed by funder, could strengths and limitations section describe what means were taken to ensure transparency of or limits on funder influence on the findings.	The following has been added to the manuscript to clarify this<br><br>“This Priority Setting Partnership was guided by a steering group made up of patient and carer representatives (n=6),five from the lived experience group and one from the Marie Curie Research Voices group, professionals from diverse health and social care backgrounds (n=9), and representatives from health and social care research funders across all UK nations (n=6). The group was chaired by a senior JLA adviser to ensure full adherence to JLA principles. Working in parallel with the steering group, the lived experience group and the JLA advisory team helped maintain transparency and rigour throughout the project.”
Page 6, Lines 14-16. "Responses could contain relate to..." sentence needs reviewing.	This has been reworded to “Responses could be linked to more than one summary question”

Page 10, line 53 review sentence "None been fully addressed by research"	This has been changed to "none had been fully addressed by research"
Page 14, line 35-38 review "Several responses to the initial survey were vague or very brief. For example, "symptom management" was detailed." - was symptom management detailed or is this an example of a vague response?	This is an example of a vague response. The sentence has been amended for clarity
**Reviewer: 2 -** Dr. Seamus Coyle, University of Liverpool	
I have no comments	
**Reviewer: 3**<br>Dr. P Chong, HCA Hospice Care, Singapore	
The section after the abstract appears to fit another journal's house style. Potentially revise to fit this journal's requirements. I recall it is about "Strengths and limitations of this study."	This has now been updated.
Under the method section, I wonder if something could be said about the James Lind Alliance approach? Figure 1 does not say much honestly. How is this different from other approaches and what are its merits, for example. This helps orient readers (like me) who may be piqued but don't have time to read the extensive guide cited. After all, this is THE study design adopted here.	Good point, the following has been added to expand upon the JLA methodology<br>"The James Lind Alliance (JLA) approach (10) was created in recognition of the fact that health research agendas have historically been shaped primarily by researchers and pharmaceutical companies. In contrast, the JLA process aims to ensure that people who are typically excluded from research prioritisation such as those with lived experience, can actively contribute. The process involves two open surveys followed by a prioritisation workshop (Figure 1). Typically, a JLA Priority Setting Partnership produces a "top 10" list of priorities that highlight important areas for future research."

Line 40/41 on page 4 is missing a full stop. At first I thought children were in, but they are excluded	This has now been updated.
Line 15/16 on page 5. Is a word missing there between “contain” & “relate”? Same for line 39/40 same page: “Two versions …” AND “piloted XXX project”?	Thanks for highlighting these errors, they have now been updated
I am wondering if Table 2 could be made easier to take in. As is, it looks like a massive spreadsheet that demands careful analysis. Shortly after comes Table 3. In contrast, that seems critical and bold fonts used help to signpost pertinent points quickly.	Table 2 contains the average ratings of each of the long-listed questions, from the perspectives of both people with lived experience and health and social care professionals. We felt that readers may be interested in where these two groups agree and/or disagree with regards to the importance of the questions identified.<br><br>The orientation of the table has been changed, so that the questions themselves are easier to read and bold font has now been used to highlight pertinent points, as has been done for Table 3. We hope these changes make the table more accessible to readers.
The discussion section appears somewhat brief. It could be expanded to explore how, where and why priorities here differ from the previous list ten years ago. Given that the procedures involved were carried out in the UK among stakeholders there, does it mean findings apply only to commissioners or clinician-researchers there?	The discussion section has been expanded, to include more comparisons of the priorities identified in 2015 and 2025. The priorities reported here are based on the views of people based in the UK, there will be relevance to international researchers, clinicians and commissioners, but it would also be interesting to explore the similarities and differences in priorities reported in different nations.
The quick mention of how questions align with the international research priorities paper by Hasson et al could be broadened too, with comments on differences, for example, given the unique methodology you adopted here.	A more detailed comparison to the international priorities identified by Hassan has been added to the discussion.

<table>
<tr><td colspan="2">Reviewer: 4
Mrs. Emma Winstanley, NHS Blood and Transplant Tissue and Eye Services</td></tr>
<tr><td>My comments relate to the limitations of the respondent groups being predominantly white women. Although this is acknowledged in the paper, this and the very broad topics, could suggest that rather than a repeat, refresh of the original, more targeted future research involving broader demographic inclusion, harder to reach groups and patients at the end of life could support these initial findings?</td><td>Thanks for this comment. As we noted in the paper, and as you highlight, one of the limitations of this work is the limited diversity among responders. We looked at the demographics of other PSP projects (where reported) and similar challenges seem to have been faced.

We felt that given 10 years had passed since the identification of the first set of priorities for palliative and end of life care in 2015, that a refresh of these was warranted, to ensure that research that is funded continues to align with the priorities of people receiving or delivering care.

Certainly, additional work is required to explore the priorities of people that are not represented within this particular project. In our recommendations for future research and the strengths and limitations sections, we have emphasised this.</td></tr>
</table>

VERSION 2 - REVIEW

**Reviewer**	**3**
**Name**	**Chong, P**
**Affiliation**	**HCA Hospice Care, Singapore**
**Date**	**21-Jan-2026**
**COI**	

Thank you for the considered responses. Looking forward to seeing this in print soon.
